# Complete Genome Sequence and Construction of an Infectious Bacterial Artificial Chromosome Clone of a Virulent Duck Enteritis Virus Strain XJ

**DOI:** 10.1155/2024/1746963

**Published:** 2024-05-17

**Authors:** Su-xin Huo, Yin-chu Zhu, Liu Chen, Tao Yun, Wei-cheng Ye, Jiong-gang Hua, Zheng Ni, Sheng-rui Xiang, Fang-zhou Ding, Xu Gao, Han-bin Liu, En-dong Bao, Cun Zhang

**Affiliations:** ^1^College of Veterinary Medicine, Nanjing Agricultural University, Nanjing 210095, China; ^2^State Key Laboratory for Managing Biotic and Chemical Threats to the Quality and Safety of Agro-products, Institute of Animal Husbandry and Veterinary Science, Zhejiang Academy of Agricultural Sciences, Hangzhou 310021, China; ^3^College of Animal Science and Technology, Zhejiang A&F University, Zhejiang 311300, China; ^4^College of Veterinary Medicine, Inner Mongolia Agricultural University, Hohhot 010018, China; ^5^Guangdong Zhongkeda Biotechnology Services Co., Ltd., Shenzhen 518000, China

## Abstract

In 2021, a highly virulent strain of duck enteritis virus (DEV), designated as DEV XJ, was isolated from Zhejiang, China, and its complete genome, spanning 162,234 bp with 78 predicted open reading frames (ORFs), was sequenced. While showing relative homology to the DEV CV strain, DEV XJ exhibited distinctions in 38 ORFs, including various immunogenic and virulence-related genes. Amino acid variation analysis, focusing on *UL6* and *LORF3*, indicated a high degree of homology between DEV XJ and the 2085 strain from Europe, as well as the DEV DP-AS-Km-19 strain from India. Subsequently, a full-length infectious bacterial artificial chromosome clone (BAC) of DEV XJ was successfully constructed to delve into the pathogenic mechanisms of this virulent strain. XJ BAC demonstrated substantial similarity to the parental DEV XJ in both *in vitro* growth properties and the induction of typical pathogenic symptoms in sheldrakes. Furthermore, the *US3*, *LORF3*, *UL21*, and *UL36* genes were individually deleted using a two-step RED recombination approach based on the infectious BAC clone. Our findings revealed that the *UL21* and *UL36* genes play crucial roles in viral proliferation. Although the *US3* and *LORF3* genes were dispensable for viral replication and cell-to-cell transmission *in vitro*, they attenuated the replication and transmission efficiency of DEV compared to the WT. In summary, this study accomplished the whole-genome sequencing of a clinically virulent DEV strain and the successful construction of an infectious DEV XJ clone. Moreover, the functional roles of the above-mentioned mutant genes were preliminarily explored through the analysis of their *in vitro* biological characteristics.

## 1. Introduction

Duck virus enteritis, also known as duck plague, is an acute, febrile, and septic infectious disease affecting various waterfowl species worldwide. This globally occurring disease is attributed to duck enteritis virus (DEV), which is referred to as *Anatid herpesvirus 1*. DEV is classified within the genus *Mardivirus* and the subfamily *Alphaherpesvirinae* of *Herpesvirinae*, according to the 2012 virus taxonomy reported by the International Committee on Taxonomy of Viruses (ICTV). Clinical symptoms of this disease include vascular damage and internal hemorrhages [1] and lesions in lymphoid organs, digestive mucosal eruption, severe diarrhea, and degenerative lesions in parenchymatous organs [2]. DEV virulence varies, with older ducks often experiencing evident clinical symptoms and high mortality rates. The disease causes significant economic losses in domestic and wild waterfowl populations. To counteract lethal DEV infections in ducks, attenuated or naturally apathogenic DEV strains are used as live vaccines, effectively preventing and controlling the disease.

Mature DEV exhibits a distinctive spherical structure characteristic of herpesviruses, boasting a diameter ranging from approximately 120 to 300 nm. It comprises linear double-stranded DNA, an icosahedral capsid, an amorphous tegument, and a bilayer lipid envelope, encapsulating a complex architecture. The complete DEV genome contains 78 ORFs predicted to encode potential functional proteins and is composed of a 5′ to 3′ orientation as a unique long (UL) region, an inverted repeat (IR) region, a unique short (US) region, and a terminal repeat (TR) region [3]. In recent years, sequences of different DEV strains have been published. Discrepancies have been identified in the size of genomes, base composition, and the arrangement of inverted and directly repeated sequences among these viral strains [4].

BACs have proven to be invaluable vectors for cloning large gene fragments. The combination of a viral BAC system and various *E. coli*-based recombination systems constitute a highly reliable and efficient method for generating a recombinant virus, exploring viral gene and protein function, and constructing gene therapy or vaccine vectors [5]. Many herpesviruses have been cloned as BAC in *E. coli*, such as Marek's disease virus (MDV) [6], Herpes simplex virus type 1 (HSV-1) [7], Human cytomegalovirus (HCMV) [8], Epstein–Barr virus (EB) [9], Pseudorabies virus (PRV) [10], and Varicella zoster virus (VZV) [11]. Wang et al. [12] first constructed the infectious viral BAC of the virulent strain 2085, and Chen et al. [13] later constructed the infectious viral BAC of the vaccine strain in our laboratory. To delve into the biology and gene functions of virulent DEV XJ strain, we constructed a full-length infectious bacterial artificial chromosome clone (BAC) of DEV XJ. In this study, we determined the complete genome sequence of DEV XJ, demonstrating that the mini-F sequence has no influence on the *in vitro* characterization and *in vivo* pathogenicity of DEV XJ.

Determining the complete genome sequence of a virulent DEV XJ strain is a crucial step in advancing research on viral variation and identification of candidate genes associated with DEV virulence. Over the years, molecular characterization of individual DEV proteins like pUL14 [14], pUL49.5 [15], and pLORF5 [16] has progressed. However, the determinants of DEV pathogenicity remain largely unknown. In particular, the DEV XJ strain examined in this research exhibits distinctions in 38 ORFs. The *US3*, *UL21*, and *UL36* genes have been shown to be associated with replication, transmission, neuroinvasiveness, and virulence in other *α*-herpesviruses,[17–19]. The *LORF3* gene is unique to avian herpesviruses and is related to their distinct biological characteristics [20–22]. In this study, we used a two-step RED recombination approach based on the infectious BAC clone to delete the *US3*, *LORF3*, *UL21*, and *UL36* genes individually, allowing for a preliminary screening and exploration of genes related to viral biological functions. The results revealed crucial roles for the *UL21* and *UL36* genes in viral proliferation, while the *US3* and *LORF3* genes attenuated the replication and transmission efficiency of DEV compared to the WT. This advancement will provide valuable insights into DEV research, shedding light on the virus's genetic makeup, variations, and potential implications for waterfowl populations.

## 2. Materials and Methods

### 2.1. Cell and Virus Strain

Duck embryo fibroblasts (DEFs) were prepared from 12-day-old specific pathogen-free (SPF) embryonated eggs and cultured in Dulbecco's modified Eagle medium (DMEM; Thermo Fisher Scientific, USA), supplemented with 10% fetal bovine serum (FBS; Thermo Fisher Scientific, USA), at 37°C in 5% CO_2_. Clinical liver tissue samples were obtained from a farm in Zhejiang province, China, in 2021. These samples were homogenized, and DEFs were subsequently infected with a supernatant containing 2% penicillin-streptomycin. The infected cells were washed with phosphate-buffered saline (PBS) and incubated in DMEM supplemented with 2% FBS. The standard highly virulent CV strain (Gene Bank accession number: JQ673560), utilized in this study, was provided by the China Institute of Veterinary Drugs Control (Beijing, China).

### 2.2. Genome Sequencing and Amino Acid Variations Analysis

The total DNA of DEV XJ and the circular, supercoiled form of the BAC clone were extracted from infected DEFs 36 hr postinfection using the SDS-proteinase K method as described previously [13]. The purified genomic DNA was sequenced through nano sequencing and Illumina sequencing (Shanghai BIOZERON Co., Ltd., Shanghai, China). The complete genome sequence of DEV XJ was annotated using Vector NTI software and deposited in the GenBank database under the accession number OR757570. Vector NTI software was performed to compare the complete sequences of DEV XJ with those of other DEV strains. Nucleotide homology and amino acid variation analyses were conducted using BLAST on the NCBI website and Snap Gene software. Amino acid sequence homology and phylogenetic trees of all DEV strains with reported genomes on the NCBI website were analyzed using the maximum-likelihood method with MEGA X software (V10.2.6).

### 2.3. PCR Identification

The materials suspected to contain DEV were homogenized, and supernatants were subsequently collected. Total DNA strands from these supernatants were extracted using the viral genomic DNA extraction kit following the instructions (GENFINE, China). The purified DNA samples were identified through polymerase chain reaction (PCR) using DEV-specific primer pairs for the *UL2* gene (Table 1). Furthermore, to verify the difference in the *LORF3* gene between DEV XJ and DEV CV, specific primer pairs of *LORF3* were designed for use in PCR (Table 1).

### 2.4. Construction of the BAC Clone of DEV XJ

The transfer vector pHA2-UL18-UL15 was provided by the Department of Poultry Diseases of the Zhejiang Academy of Agriculture Science. The transfer vector was prepared according to the instructions of the Plasmid Mini Kit I (Omega, USA). Then, the circular plasmid was digested with *Hind III*, and the linearized fragment was obtained following the protocol of the MiniBEST DNA Fragment Purification Kit Ver.4.0 (TAKARA, China). On the basis of Chen et al. [13], the linearized plasmid was recombined with DNA of DEV XJ. The recombinant virus was obtained after 10 cycles of plaque picking and selection medium screening. The selection medium comprised 300 *μ*g/mL mycophenolic acid (Sigma, USA), 60 *μ*g/mL xanthine (Sigma, USA), 100 *μ*g/mL hypoxanthine (Sigma, USA), 2% FBS, and 1% penicillin-streptomycin. The DNA of the recombinant virus was extracted and electroporated into *E.coli* DH10B competent cells (Thermo Fisher Scientific, USA). Positive BAC clone was obtained through chloramphenicol-resistant screening. Restriction endonuclease*BamHI*, *BhlII*, *EcoRV*, and *XbaI* were used for restriction fragment length polymorphism (RFLP) identification. The correct BAC DNA was then transfected into DEFs using calcium phosphate precipitation according to Morgan et al. [23], and a stable inheritable recombinant virus named XJ BAC (Gene Bank accession number: PP408247) was generated.

### 2.5. Genetic Stability of XJ BAC *In Vitro*

DEFs cells were separately infected with different passages of XJ BAC, and viral harvests were collected 72 hr postinfection. Samples were processed with a Tissuelyser-24 at 60 Hz for 90s and centrifuged at 5,000 rpm before titration. Viral titers were determined by calculating the 50% tissue culture infectious dose (TCID_50_), and statistical analyses were conducted using GraphPad Prism version 9.4.0. All experiments were performed and repeated in triplicate.

### 2.6. Growth Kinetics of XJ BAC *In Vitro*

DEFs cells in 12-well plates were infected with 0.02 MOI of XJ BAC and DEV XJ. Cell and culture supernatant samples were collected at 0, 12, 24, 36, 48, 72, 96, and 120 hr postinfection and stored at −80°C until the completion of sample collection. Samples were treated with Tissuelyser−24 at 60 Hz for 90s and centrifuged at 5,000 rpm for 5 min before titration. Intracellular and supernatant viral titers were detected by determining the TCID_50_, and multistep growth curves were constructed based on data from three independent experiments. Statistical analyses were performed using GraphPad Prism version 9.4.

### 2.7. The Plaque Morphology of Recombinant Viruses

The cell-to-cell spread function of DEV XJ and XJ BAC was measured through plaque size assays. DEFs in 12-well plates were infected with specified viruses at an MOI of 0.002. Following a 2-hour incubation at 37°C, DMEM containing 1% agarose with low gelling temperature was added to cover the infected cells. After a 6-day incubation at 37°C, the cells were fixed with 4% paraformaldehyde and stained with 1% crystal violet. A total of 100 randomly selected plaques were chosen for each virus, and their plaque sizes were measured using Image J software. Statistics analyses were performed using GraphPad Prism version 9.4. Data were considered not significantly different if the *P* value was > 0.05 (ns, *P* > 0.05). All reactions were performed and repeated in at least three independent experiments.

### 2.8. Electron Microscopy Analysis of Recombinant Virus

DEFs cultured in TC Flask T75 were infected with DEV XJ and XJ BAC at an MOI of 2. Infected cells were scraped, collected, and centrifuged at 1,500 rpm for 10 min. The supernatant was then discarded, and the cells were fixed with fixative solution (HAOKEBIO, China). Samples were sent to HaoKe Biotechnology Co., Ltd. for analysis under a transmission electron microscope.

### 2.9. *In Vivo* Experiment

To investigate the potential impact of mini-F on the pathogenesis and lethality of DEV XJ, animal experiments were performed. 60-day-old sheldrakes, confirmed to be free of DEV and DEV antibodies, were intramuscularly injected with 10^4^ TCID_50_ of DEV XJ, XJ BAC, and DMEM for viral rejuvenation. Then, the livers of infected ducks were collected and homogenized, and the viral content was determined using qPCR. Another set of 60-day-old sheldrakes, also confirmed to be free of DEV and DEV antibodies, was intramuscularly injected with 10^6^ DNA copies of liver supernatant of DEV XJ (*n* = 10), XJ BAC (*n* = 10), and DMEM (*n* = 10) groups. The ducks were monitored daily, and those that succumbed to the disease or were humanely euthanized were examined for histopathology.

### 2.10. Quantification of DEV Genome Copies

The liver, thymus, brain, and intestines were taken from the infected animals at 1, 3, and 5 dpi of each group to assess virus replication *in vivo*. Total DNA strands of the supernatants were extracted using a viral genomic DNA extraction kit according to the instruction (GENFINE, China). THUNDERBIRD Probe qPCR Mix (TOYOBO, China) was used to quantify viral DNA copies. The primers and probe for detecting the DEV *UL44* gene by qPCR had been previously designed in our laboratory. qPCR amplifications were carried out with the following conditions: 95°C for 60 s, followed by 40 cycles at 95°C for 15 s and 60°C for 45 s. The result of the qPCR was quantified by comparison with the established curve. All reactions were performed in triplicate and repeated in at least three independent experiments.

### 2.11. Construction of Mutant Strains Using Two-Step RED-Mediated Recombination

In this study, we employed US3-deleted mutant (XJ-*Δ*US3) and LORF3-deleted mutant (XJ-*Δ*LORF3), which were constructed through two-step RED-mediated recombination. First, *Δ*US3-Kan-F/R and *Δ*LORF3-Kan-F/R primers (Table 1) were utilized to amplify *Δ*US3-Kan and *Δ*LORF3-Kan fragments via PCR, respectively. During the initial step, the target genes were replaced with the kanamycin resistance gene. Subsequently, in the second step of homologous recombination, the I-Sec I site was cleaved, and kanamycin fragment was excised, resulting in the desired deletion. For PCR amplification, *Δ*US3-F/R and *Δ*LORF3-F/R primers were employed. Following successful amplification, plasmids from the positive BAC clones were transfected into DEFs using calcium phosphate precipitation, facilitating the generation of the recombinant virus.

## 3. Results

### 3.1. Isolation of DEV Strain XJ

**Figure 1:**
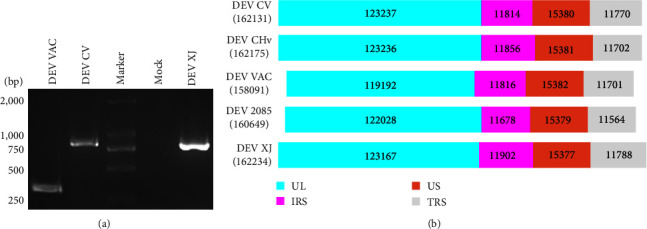
Isolation of DEV XJ strain and genomic organization of DEV. (a) Identification of isolated virus by PCR analysis. (b) Lengths of various regions in the DEV genome.

The liver tissue from suspected duck enteritis virus (DEV) positive sheldrakes was identified using the PCR method, indicating the presence of the DEV *UL2* gene, with PCR fragments measuring 838 bp in length—consistent with DEV virulent strains CV and CHv, as opposed to the 310 bp observed in the vaccine strain DEV VAC (Figure 1(a)). Tissue supernatants were incubated in duck embryo fibroblasts (DEFs). The purified DEV was successfully obtained after three rounds of plaque purification, and it was named as DEV XJ.

### 3.2. Genomic Organization of DEV XJ

To reveal the genetic characteristics of the DEV XJ, the complete genome sequence was determined to be 162,234 bp in length using high-throughput sequencing. A total of 76 annotated ORFs of the CV strain were identified in the XJ strain, with no additional ORFs observed. The GC content of DEV XJ was found to be 45%, consistent with other published DEV strain. The unique long (UL) region of DEV XJ from nucleotides (nt) position 1–123167, exhibiting a shorter length compared to DEV CV due to deletion within the UL coding region. The unique short (US) region extends from nt position 135069 to 150446, differing by only 3 bp from DEV CV. The internal inverted repeat sequences (IRS) and terminal inverted repeat sequences (TRS) flanking the US region display two discontinuous deletions and two discontinuous additions within the noncoding inverted repeats (Figure 1(b)). The impact of these deletions and additions on the characteristics of the virus is yet to be established.

### 3.3. Amino Acid Variations Analysis of the DEV XJ

**Figure 2:**
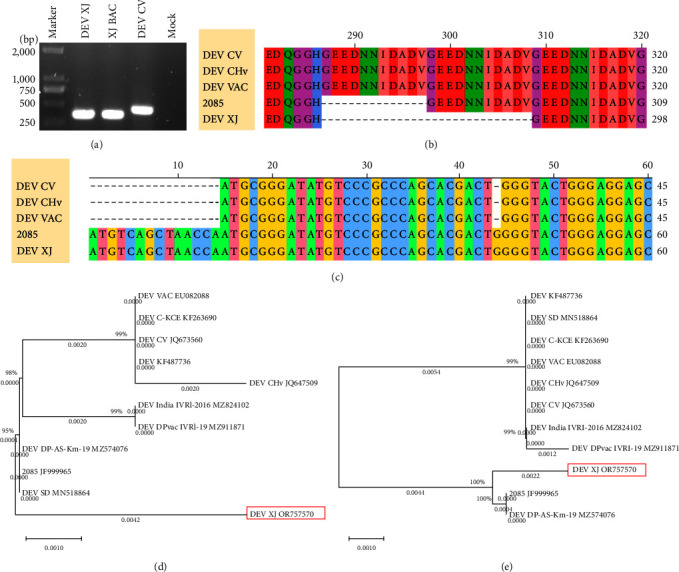
Amino acid variations analysis of the DEV XJ. (a) Identification of 22-aa deletion in LORF3 of DEV XJ by PCR analysis. (b) Amino acids deletions in LORF3. The ORFs are shown with the start codon on the left and the stop codon on the right side. Lengths and sites of amino acids in ORF are marked. (c) The frameshift mutations in UL6 of DEV XJ resulted from an extra guanosine. (d) A phylogenetic tree that is based on amino acid sequences of LORF3. The virus strain in red boxed areas indicates the DEV isolated in this article. (e) A phylogenetic tree that is based on amino acid sequences of UL6. The virus strain in the red rectangle indicates the DEV isolated in this article.

To identify nucleotide and amino acid variations, all available ORFs were compared with NCBI BLAST or Vector NTI. The 38 ORFs in the XJ strain differed from the CV strain, encompassing 46 single amino acid variations, two single amino acid deletions, and one 22-amino acid deletions (Table S1). A notable amino acid variation was observed in LORF3, which exhibited a 22-aa deletion compared to the CV, CHv, and VAC strains (Figure 2(b)). To rule out potential issues arising from gene splicing and assembly in high-throughput sequencing, this result was further confirmed by PCR (Figure 2(a)). A similar deletion has also been noted in the case of the 2085 strain, which lacks 11-aa, constituting one repeat in LORF3. For UL6 ORF, the XJ strain's UL6 sequence displayed an additional guanosine at position 45 compared to the CV, CHv, and VAC strains, leading to an alteration of the initiation site (Figure 2(c)). Although it has an ATG-starting at position 15, sequence analysis showed that the guanosine insertion would lead to premature termination of translation if translation initiates from this position. The same change was observed in the 2085 strain, and this change does not influence the functional impairment or inactivation of the protein in both strains by sequence researching [12]. Phylogenetic trees based on the amino acid sequences of LORF3 and UL6 were constructed and analyzed for these variations. Although the LORF3 protein of DEV XJ exhibited 97.23% highest homology with the 2085 strain, their evolutionary relationships placed them in different branches (Figure 2(d)). The UL6 protein of DEV XJ displayed 99.75% highest homology with the 2085 strain and the DEV DP-AS-Km-19 strain (GeneBank ID: MZ574076), establishing the closest evolutionary relationship with these two strains (Figure 2(e)).

To assess variations of major immunogenic and virulence-related genes, the homology ratio of glycoproteins, including gB, gC, gD, gE, gG, gH, gI, gJ, gK, gL, gM, and gN, as well as the virulence protein TK, was compared across different strains using NCBI BLAST (Table 2). The result showed distinct homology levels for various glycoproteins: gB (99.90%), gC (99.77%), gE (99.39%–100.00%), gG (99.78%–100.00%), gI (97.40%–100%), gJ (99.5%–99.81%), and gM (99.02%–99.90%), when compared to the other four strains. gK, gN, gH, gD, and TK exhibit 100% homology across all five strains, indicating stability transcending geographic boundaries as European (2085) and Asian strains specify identical or nearly identical nucleotide sequences.

### 3.4. Construction of Recombinant Virus of DEV XJ

**Figure 3:**
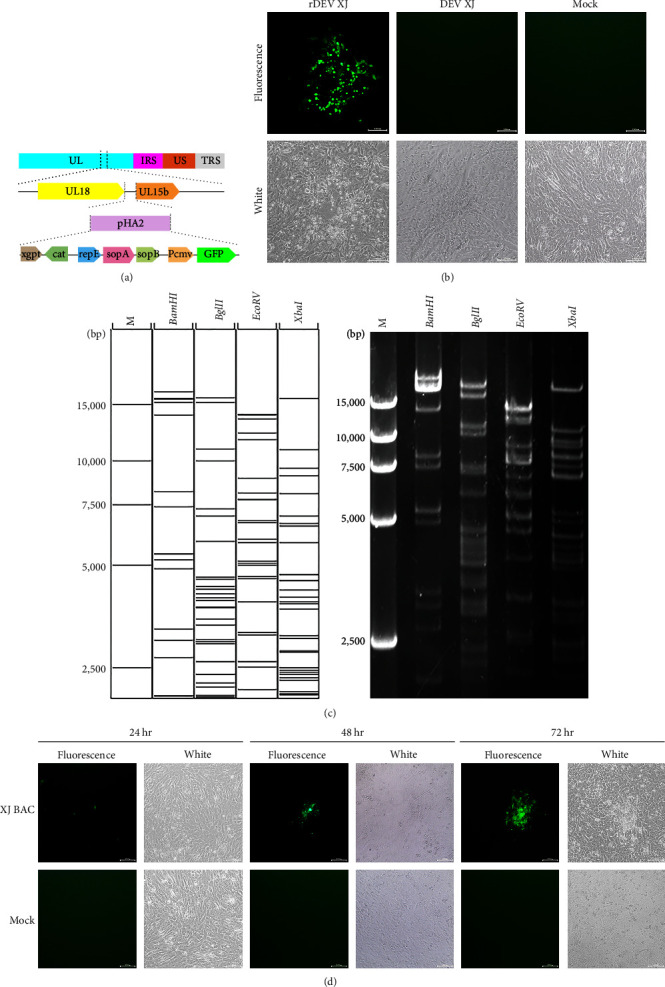
Construction of recombinant virus XJ BAC. (a) Schematic drawing of the cloning procedure to introduce the pHA2 vector into the DEV XJ genome. (b) Homogeneous population of recombinant virus rDEV XJ in DEFs. (c) Restriction fragment analysis of full-length BAC clone pDEV-XJ. The left is the prediction based on the complete DEV XJ genome by using Vector NTI. (d) Rescue of the recombinant virus XJ BAC. Plasmids from a positive colony were transfected into DEFs by calcium phosphate, and with continuous observation, the XJ BAC fluorescent marker protein EGFP was expressed in DEFs.

To elucidate characteristics of DEV XJ, a full-length infectious bacterial artificial chromosome (BAC) clone of DEV XJ was generated. DEFs were transfected with the linearized transfer vector pHA2-UL18-UL15 and then infected with DEV XJ. Purified recombinant rDEV XJ, carrying mini-F sequences flanked by loxP sites inserted within the noncoding region between UL18 and UL15 without deletion of any viral sequence, was obtained after 10 rounds of mycophenolic acid screening, plaque picking, and endpoint dilutions passing of recombinant progeny virus identified by GFP expression (Figures 3(a) and 3(b)). Circular viral DNA extracted from rDEV XJ was subsequently electroporated into *E.coli*. A positive BAC clone was acquired through chloramphenicol-resistant screening and confirmed by PCR amplification and restriction fragment length polymorphism (RFLP) analysis (Figure 3 (c)). The DNA of selected colonies was extracted and digested with *BamHI*, *BhlII*, *EcoRV*, and *XbaI*, and the RFLP results indicated that the complete identity of the bands from the colonies was compared with the predicted results.

The infectious clone plasmids were extracted and transfected into DEFs using calcium phosphate precipitation to generate the recombinant virus XJ BAC. A typical cytopathic effect (CPE) with green fluorescence was observed in DEFs on the third day (Figure 3 (d)). The recombinant virus of DEV XJ (XJ BAC) demonstrated stable passage in new DEFs, affirming the successful rescue of the recombinant virus. During the passages, the viral titers of XJ BAC remained around 10^4.8^ TCID_50_/0.1 mL, with analysis indicating no significant differences between generations, demonstrating stable titers throughout the recombinant virus passages (Figure S1).

### 3.5. Mini-F Sequence Has No Influence on the Viral Growth Kinetics

**Figure 4:**
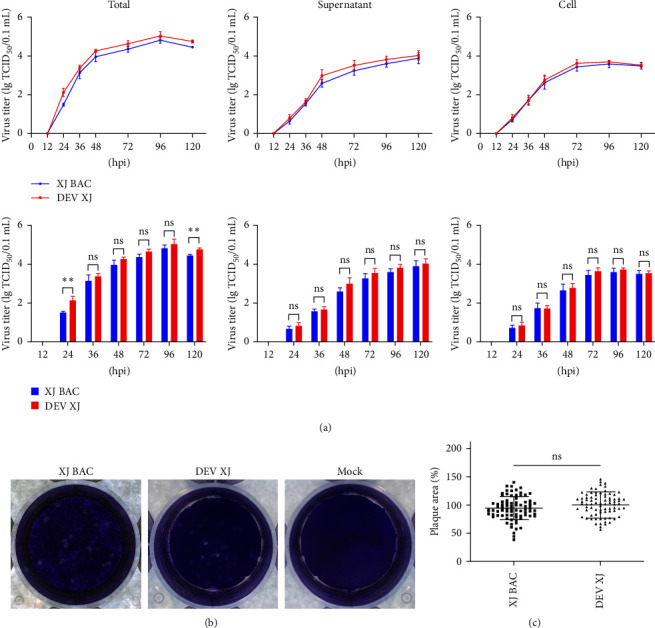
Comparison of biological characteristics of DEV XJ and XJ BAC *in vitro*. (a) Multistep growth of DEV XJ and XJ BAC. The viral titers of infected cells were determined at different times after inoculation of approximately 0.02 MOI of viruses. Statistical analysis of the differences in virus titers in the total, cell, and supernatant at each time point. The multistep growth curves were computed from three independent experiments. (b) Plaque of DEV XJ and XJ BAC. (c) The viral plaque size was measured by Image J. Representative plaques showing 80 plaques per sample were measured to quantify the results (ns, *P* > 0.05;  ^*∗∗*^, *P* < 0.01).

To explore the growth kinetics of the recombinant virus and its parental virus, the multistep growth kinetics of the DEV XJ and XJ BAC were compared *in vitro*. Growth kinetics data revealed that the recombinant virus XJ BAC exhibited growth characteristics nearly identical to the parental virus DEV XJ (Figure 4(a)). Between 12 and 96 hr postinfection, both viruses showed the same replication pattern, with viral titers steadily increasing and reaching their peak at 96 hr. Subsequently, virus replication gradually decelerated, then reaching a plateau. These findings suggested that the insertion of the mini-F sequence into the genome of DEV XJ has no discernible impact on the growth kinetics of XJ BAC.

The function of the virus spreading from cell to cell was investigated using plaque size assays. The 1% agarose with a low-gelling temperature solid cell culture medium was used to ensure that neighboring cells were exclusively infected through viral cell-to-cell transmission. The cytopathic plaque of two viruses was measured by crystal violet staining after DEFs were infected for 6 days (Figure 4(b)). Statistics analysis was performed using Image J and GraphPad Prism 9.4.0, revealing that the plaque areas of the recombinant virus XJ BAC are 5.85% smaller than the parental virus DEV XJ. However, no significant differences were observed between two groups (*P*=0.0957) (Figure 4(c)). The results demonstrated that the insertion of mini-F sequence had only a slight effect on the viral plaque size.

### 3.6. Mini-F Sequence Has No Influence on the Viral Morphogenesis

To investigate the potential impact of mini-F insertion on viral morphogenesis, virions at various morphogenetic stages were examined using transmission electron microscopy (TEM) (Figure S2). Capsid assembly occurs in the nucleus during the DEV proliferation. The particles in the nucleus of DEV XJ and XJ BAC exhibited a similar structure, with the electron-dense core enclosed within capsids (Figure S2A-a and S2D-a). Viral envelopment of DEV occurs in the cytoplasm. The electron micrographs displayed complete and regular viral particles, with no discernible differences in virion structure between the two viruses (Figure S2(C-b) and S2(D-b)). Additionally, empty capsids resulting from incorrect assembly were observed in both the nucleus and cytoplasm of DEV XJ and XJ BAC (Figure S2(A-d) and S2(D-d)). Subsequently, complete virions were transported out of cells by entering vesicles. Released viral particles, with a diameter of approximately 300 nm for both DEV XJ and XJ BAC, exhibited a more mature structure and high electron density at this stage (Figure S2(C-c) and S2(F-c)). These results indicated that mini-F sequences do not play a role in the viral morphogenesis of DEV XJ, including viral assembly in the nucleus and viral envelopment in the cytoplasm.

### 3.7. The Pathogenesis and Lethality of DEV XJ and XJ BAC

**Figure 5:**
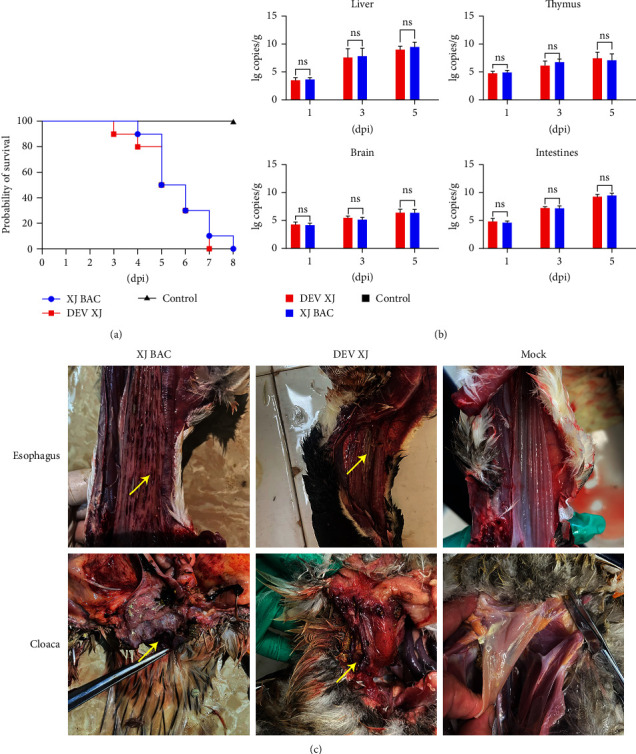
Pathogenesis of DEV XJ and XJ BAC. (a) Survival curve of sheldrakes infected with DEV XJ (*n* = 10) and XJ BAC (*n* = 10). (b) Replication characteristics of DEV XJ (*n* = 15) and XJ BAC (*n* = 15) *in vivo* (ns, *P* > 0.05). (c) Autopsy histopathology of the dead sheldrakes. The lesion of the organ was pointed by a yellow arrow.

To estimate the pathogenicity and lethality of the DEV XJ strain and XJ BAC strain, 60-day-old sheldrakes, confirmed to be free of DEV and DEV antibodies, were intramuscularly injected with liver supernatants of DEV XJ, XJ BAC, and DMEM. The result showed that the mortality curve of sheldrakes infected with XJ BAC exhibited a similar trend to that of DEV XJ (Figure 5(a)). Sheldrakes succumbed to the infection between the third or fourth day and the seventh or eighth day postinfection, with a rapid increase in mortality observed on the fifth and sixth days. Autopsy results of the deceased sheldrakes demonstrated typical DEV symptoms in the tissue (Figure 5(c)), including bleeding spots and pseudomembrane on the esophagus surface in both DEV XJ and XJ BAC groups. Additionally, the bleeding spot was observed on the cloacal surface in two experimental groups. Viral replication in the tissue organs of infected sheldrake was determined by qPCR at various time points postinfection. The results showed that replication of XJ BAC in the liver, thymus, brain, and intestines did not exhibit significant differences compared to that of DEV XJ (*P*  > 0.05) (Figure 5(b)). These results collectively indicated that the insertion of mini-F sequences does not play a role in the pathogenesis and lethality of DEV XJ.

### 3.8. Construction of Recombinant Viruses

**Figure 6:**
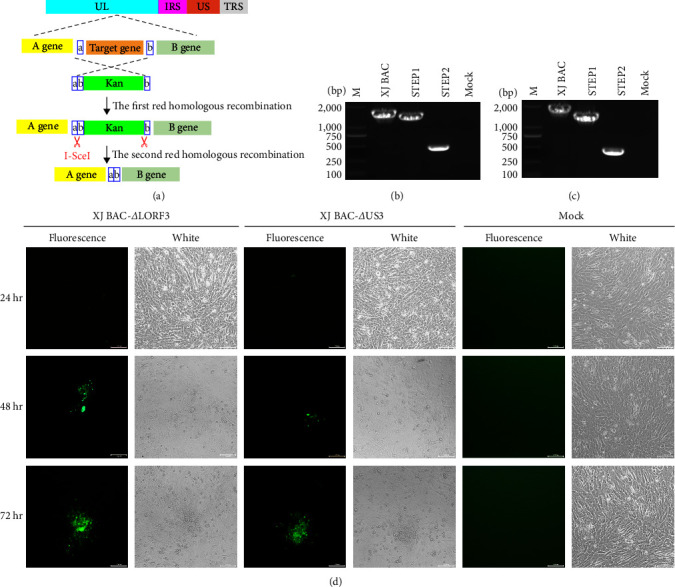
Construction and identification of recombinant viruses. (a) Schematic representation of the two-step RED-mediated recombination process for constructing the US3-deleted mutant and LORF3-deleted mutant. (b) PCR identification of the two-step RED-mediated recombination for the US3-deleted mutant. (c) PCR identification of two-step RED-mediated recombination for the LORF3-deleted mutant. (d) Rescue of recombinant viruses.

To investigate the potential impact of mutations, the *US3*, *LORF3*, *UL21*, and *UL36* genes were individually deleted based on the infectious BAC clone using a two-step RED recombination approach. The results revealed that pXJ BAC-*Δ*UL21 and pXJ BAC-UL36 were unrescuable (data were not exhibited). Positive BAC clones for pXJ BAC-*Δ*US3 and pXJ BAC-*Δ*LORF3 were validated through PCR amplification and subsequent sequencing (Figures 6(b) and 6(c)). The PCR product for the US3-deleted and LORF3-deleted strains were 425 bp and 450 bp, respectively. Following this confirmation, the positive BAC clones were transfected into DEFs using calcium phosphate precipitation, resulting in the successful generation of recombinant viruses XJ BAC-*Δ*US3 and XJ BAC-*Δ*LORF3 (Figure 6(d)).

### 3.9. *US3* and *LORF3* Affect the Replication and Transmission of DEV *In Vitro*

**Figure 7:**
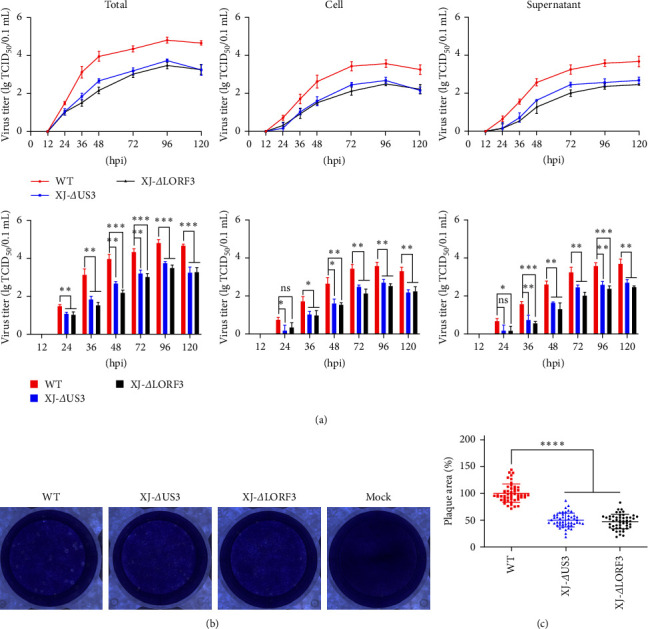
Comparison of biological characteristics of XJ-*Δ*US3, XJ-*Δ*LORF3, and WT *in vitro*. (a) Multistep growth kinetics of XJ-*Δ*US3, XJ-*Δ*LORF3, and WT. Viral titers of infected cells were assessed at different time points after inoculation with approximately 0.02 MOI of viruses. Statistical analysis of the differences in virus titers in the total, cell, and supernatant at each time point. Multistep growth curves were generated from three independent experiments. (b) Plaque morphology of XJ-*Δ*US3, XJ-*Δ*LORF3, and WT. (c) Quantification of viral plaque size using Image J. Representative plaques from each sample (50 plaques per sample) were measured to quantify the results (ns, *P* > 0.05;  ^*∗*^, *P* < 0.05;  ^*∗∗*^, *P* < 0.01;  ^*∗∗∗*^, *P* < 0.001; and  ^*∗∗∗∗*^, *P* < 0.0001).

To investigate the roles of *US3* and *LORF3* genes in replication, multistep growth kinetics assays were conducted (Figure 7(a)). The growth curve of XJ-*Δ*US3, XJ-*Δ*LORF3, and wild-type (WT, DEV XJ) viruses revealed a similar replication pattern between 12 and 96 hr postinfection, with viral titers steadily increasing and peaking at 96 hr. Subsequently, virus replication gradually decelerated, reaching a plateau. Data differences were analyzed using GraphPad Prism 9.4.0, showing significantly reduced titers of mature viral particles in supernatants, cells, and whole smples after infection with the XJ-*Δ*US3 and XJ-*Δ*LORF3 compared to the WT. Growth kinetics revealed that although the *US3* and *LORF3* genes were dispensable for viral replication *in vitro*, they attenuated the replication efficiency of DEV compared to the WT.

The function of the virus spreading from cell to cell was investigated using plaque size assays. Compared with the WT group, the plaque areas were significantly reduced after infection with XJ-*Δ*US3 and XJ-*Δ*LORF3 (Figure 7(b)). Subsequently, 50 plaques were randomly selected from each group, and their size was measured using Image J software, followed by statistical analysis conducted with GraphPad Prism 9.4.0. The results revealed that the plaque areas of the recombinant virus XJ-*Δ*US3 were 49.69% smaller than those of the WT, while the plaque areas of the recombinant virus XJ-*Δ*LORF3 were 52.63% smaller than the WT (Figure 7(c)). These findings underscored the impact of *US3* and *LORF3* on the function of virus spreading from cell to cell.

## 4. Discussion

Duck plague, caused by the duck enteritis virus (DEV), is a severe infectious disease affecting waterfowl at various ages. Natural outbreaks have been reported from 7 days of age to adulthood, resulting in huge economic losses for the global waterfowl industry [24]. Despite studies on the pathogenesis and immunological mechanisms of virulent and attenuated forms of DEV [25, 26], many genes still play crucial roles in the pathogenic process. The horizontal transmission of DEV from migratory waterfowl, acting as carriers during outbreaks, may contribute to viral variation. Although convalescent birds may develop resistance to reinfection, they can become carriers, shedding the virus into the environment for an extended period [27]. Therefore, diverse clinical strains were isolated, and genomic analysis may provide more valuable molecular characteristics, enhancing our understanding of the evolution of DEV strains.

In this study, the complete genomic sequences of the DEV XJ strain were elucidated using nano sequencing and Illumina sequencing methods. The genome structure primarily follows the 5′ to 3′ orientation, resembling other published DEV strains, with the order UL-IRS-US-TRS. Sequencing results revealed major amino acid variations in DEV XJ, notably in the LORF3 protein, which has a 22-aa deletion, and the UL6 proteins, where the initiation site has an alteration compared to the DEV CV strain. Despite sharing a high homology of 99.93% with the DEV CV strain, both the LORF3 and UL6 proteins in DEV XJ exhibit significant homology with the 2085 strain isolated in Europe [28] and the DEV DP-AS-Km-19 strain isolated in India [29]. This suggests potential horizontal transmission from migratory waterfowls, given their reliance on aquatic environments for survival. The susceptible population may be exposed through direct contact with infected migratory waterfowls and indirectly through the virus-contaminated environment. Thus, both host factors and transmission dynamics may contribute to virus variation.

In addition, there is limited knowledge about the molecular mechanism of lethality, and neurovirulence, which is closely associated with latent infection. While several studies have reported the virulence of DEV, but little is known in this regard. Thus, reverse genetics is a powerful tool for pathogenisis studies and the development of an effective vaccine. Various strategies exist for viral reverse genetics methods, with a full-length infectious viral BAC clone being an invaluable tool compared with the overlapping fosmid DNA system. BACs have a large capacity and are commonly utilized to clone DNA fragments ranging from 150 to 300 kb. Viral genomes have stable maintenance and inheritance in *E. coli*, and BACs enable easy generation of various modifications to the viral genome using different *E. coli*-based recombination systems. To enhance the co-transformation efficiency, several new rapid methods for generating infectious BAC clones have been proposed. Yuan et al. [30] directly cloned the PRV genome into a BAC in *E. coli* using Exonuclease Combined with RecET recombination (ExoCET). Knickmann et al. [31] developed a method based on single-step transformation-associated recombination (STAR) in *Saccharomyces cerevisiae*. Zhou et al. [32] introduced a novel approach by combining highly efficient Tn5-based *in vitro* transposition with the high capacity of the BAC vector. These diverse and efficient methods of BAC construction offer researchers selective opportunities to investigate various herpesviruses. Building on the methodology used in constructing the DEV VAC BAC from our laboratory [13], where the insertion of foreign genes into the intergenic region between genes *UL15* and *UL18* in the DEV vaccine strain proved stable and had no discernible impacts on viral growth *in vitro*, the same location was chosen for the construction of an infectious viral BAC for DEV XJ in this study. The result shows the successful construction of the infectious DEV XJ clone. The insertion of the mini-F sequence had a minor effect on viral cell-to-cell transmission but did not influence the viral replication *in vitro*, as evidenced by measurements of plaque area and multistep growth kinetics. Moreover, according to the results of the animal regression test, the insertion of the mini-F sequence is not implicated in the pathogenesis and lethality of DEV XJ.

Subsequently, the *US3*, *UL21*, *LORF3*, and *UL36* genes, which exhibit distinctions in DEV XJ strain, were individually deleted using a two-step RED recombination approach based on the infectious BAC clone. This enabled a preliminary screening and exploration of genes related to viral biological functions. pUL21 is an inner tegument protein of PRV known to promote retrograde transport in neuroinvasion, with its carboxyl terminus contributing to viral neuroinvasion [19]. pUL36 is the largest protein encoded by HSV-1 and resides in the innermost layer of the viral tegument, playing multiple roles in the HSV-1 life cycle, including capsid envelopment [18]. pUS3 is a viral serine/threonine kinase conserved among all members of the *α*-herpesvirus family and is associated with increased intercellular virus spread [17]. pLORF3 is unique to avian herpesviruses and is related to their distinct biological characteristics [33]. In this article, the *UL21* and *UL36* genes showed crucial roles in viral proliferation, while the *US3* and *LORF3* genes impacted replication and transmission efficiency. The viral replication cycle involves adsorption, invasion, viral nucleic acid replication, assembly of capsids, maturation, and virus release [34]. Further verification is needed to determine the specific functions of these four genes in the viral life cycle and how they regulate viral replication. pUL36 and pUL21 are important structural proteins of herpesviruses. Currently, gene-deleted mutants of UL36 and UL21 have only been reported in mammalian herpesviruses and not in avian herpesviruses [18, 35]. In this study, attempts to rescue UL21-deleted and UL36-deleted mutants of DEV XJ were unsuccessful, which may be attributed to species differences. The *LORF3* gene comprises three repetitive sequences, with one repeat exhibiting a 5′ to 3′ orientation as Gly-Glu-Glu-Asp-Asn-Asn-Ile-Asp-Ala-Asp-Tyr. Upon examination of sequences published on the NCBI website, it was found that the 2085 strain, SD strain, and DP-AS-Km-19 strain each have a deletion of one repeats at this location. Notably, the XJ strain displays a previously unreported deletion of two repeats, adding a unique aspect to the genetic makeup of DEV *LORF3*. Further verification is required to determine whether this deletion will influences the function of the DEV *LORF3* gene.

In conclusion, this study conducted whole-genome sequencing of the newly isolated virulent strain DEV XJ and generated a recombinant virus XJ BAC with similar biological characteristics to the parental strain. The function of *US3*, *UL21*, *LORF3*, and *UL36* genes was preliminarily screened and explored. Our data provide a foundation for further evaluation of the molecular mechanisms and variations of the duck enteritis virus.

## 5. Conclusion

The new duck plague strain discussed in this article originated from a severe outbreak on a farm in Zhejiang province, China, in 2021. Compared to the classical duck plague virus strain CV in China, the XJ strain exhibits mutations in 38 ORFs, including 46 amino acid changes, two single amino acid deletions, one 22-amino acids deletion, and a frameshift mutation. These mutations involve various immunogenic and virulence-related genes, with some being previously reported and others, like the deletion of two repetitive sequences in *LORF3*, being novel. The impact of these mutations on the virus's epidemiological characteristics remains unclear and requires further investigation. To delve into the biology and gene function of DEV XJ strain, a full-length infectious bacterial artificial chromosome clone of DEV XJ was constructed. The recombinant virus XJ BAC demonstrated substantial similarity to the parental DEV XJ in both *in vitro* growth properties and the induction of typical pathogenic symptoms in sheldrakes. Subsequently, the study investigates the potential impact of mutations by individually deleting the *US3*, *LORF3*, *UL21*, and *UL36* genes based on the infectious BAC clone using a two-step RED recombination approach. The *UL21* and *UL36* genes showed crucial roles in viral proliferation, while the *US3* and *LORF3* genes impacted replication and transmission efficiency. Reverse genetics serves as a potent tool for pathogenicity studies and vaccine development. This research will provide valuable data for DEV study.

## Figures and Tables

**Table 1:** Primers used in this article.

Primer	Sequence (5′ - 3′)
UL2-F	TGACCAACCAACGTCTACATGC
UL2-R	CAGAAAGCCTTAAATTCAGCGTG
LORF3-F	CATTACTATACAACACGGCACG
LORF3-R	GGGCAACAGAAACAGGGA
UL18-F	AAGGACCGCCTACTATCAAA
UL15-R	AAACGGGTGGCTGTGCTGAT
EGFP-F	TACGTCCAGGAGCGCACCATCT
EGFP-R	TCTTCTGCTTGTCGGCCATGATAT
*Δ*US3-Kan-F	AACAAACATACAAAACTGCCGCGGACGCAGCTCAAATGAAATTACAATTAATATATGGGAGGATGACGACGATAAGTAGGGA
*Δ*US3-Kan-R	TTTTTTACAAACTTGACTCCTCCCATATATTAATTGTAATTTCATTTGAGCTGCGTCCGCGGGTAATGCCAGTGTTACAACC
*Δ*US3-F	CGTCTCCTCCTTATGCTG
*Δ*US3-R	CCTTGCTGCGGCTATCTC
*Δ*LORF3-Kan-F	ACAGAGGACGGCCTAGCCAAGAAGCCAGACGAACTACGCACAGCTTATGGGTAACAGGCGACCTAGGATGACGACGATAAGTAGGGA
*Δ*LORF3-Kan-R	ATTACTAACCCCCTCTTTTATGTCCTAGGTCGCCTGTTACCCATAAGCTGTGCGTAGTTCGTCTGGGGTAATGCCAGTGTTACAACC
*Δ*LORF3-F	GGTCTGGTGGTCGTTTAGT
*Δ*LORF3-R	AAATCGTATCCCGTGAGTA

**Table 2:** Amino acid homology of major immunogenic and virulence-related proteins.

Gene	Protein	Length of aa	Homology ratio (%)			
			DEV CV	DEV CHv	2085	DEV VAC
*UL53*	gK	343	100	100	100	100
*UL49.5*	gN	95	100	100	100	100
*UL44*	gC	431	99.77	99.77	99.77	99.77
*UL27*	gB	1000	99.9	99.9	99.9	99.9
*UL22*	gH	834	100	100	100	100
*UL10*	gM	409	99.51	99.51	99.02	99.26
*UL1*	gL	236	100	100	100	99.58
*US4*	gG	459	100	100	99.78	100
*US5*	gJ	539	99.81	99.81	99.5	99.81
*US6*	gD	422	100	100	100	100
*US7*	gI	371	100	100	99.73	97.4
*US8*	gE	490	100	100	99.39	100
*UL23*	TK	358	100	100	100	100

## Data Availability

All the data generated during this study are included in the manuscript. Additional data related to this article may be requested from the corresponding authors.

## Supplementary Materials

Supplementary Materials Table S1: amino acid variations of DEV XJ strain compared with DEV CV strain. Figure S1: genetic stability of recombinant virus XJ BAC. Figure S2: ultrastructural morphologies of DEV XJ and XJ BAC. DEFs were infected with approximately 0.02 MOI of viruses and examined by electron microscopy.
