# Supplementary material for: Complete Genome Sequence and Construction of an Infectious Bacterial Artificial Chromosome Clone of a Virulent Duck Enteritis Virus Strain XJ
Source: Transbound Emerg Dis. 2024 May 17;2024:1746963. doi: 10.1155/2024/1746963 (PMC12016869; doi:10.1155/2024/1746963)
Supplement: Supplementary Materials — Table S1: amino acid variations of DEV XJ strain compared with DEV CV strain. Figure S1: genetic stability of recombinant virus XJ BAC. Figure S2: ultrastructural morphologies of DEV XJ and XJ BAC. DEFs were infected with approximately 0.02 MOI of viruses and examined by electron microscopy. [file 1746963.f1.docx]

| **Table S1.** Amino acid variations of DEV XJ strain compared with DEV CV strain. | | |
| --- | --- | --- |
| **ORF** | **Number (aa)** | **Amino Acid Residues and Position** |
| LORF11 | 2 | T91A S183T |
| UL54 | 2 | C429Y Q433R |
| UL52 | 1 | L189M |
| UL47 | 3 | R46C V73I V503I |
| UL46 | 2 | I110V G738W |
| UL44 | 1 | D74N |
| UL43 | 1 | M74T |
| UL42 | 1 | E209G |
| UL39 | 1 | Y6H |
| UL36 | 6 | G1362E V1860I Q1982H Y2481D G3167D S3334P |
| UL29 | 1 | P887S |
| UL27 | 1 | R674H |
| UL25 | 2 | V411I Q558H |
| UL21 | 1 | G254E |
| UL19 | 2 | Q115R S929R |
| UL14 | 1 | V70I |
| UL10 | 2 | A213T M339V |
| UL9 | 1 | D280Y |
| UL7 | 2 | K40E D231N |
| UL6 | 2 | S379T Q746R |
| UL5 | 1 | G802R |
| UL4 | 1 | 175(ΔE) |
| UL3 | 1 | V38G |
| LORF3 | 4 | 278-308(ΔGEEDNNIDADVGEEDNNIDADV) D307E R391H N442T |
| IRS | 1 | P327H |
| US1 | 1 | D102N |
| US10 | 1 | A132V |
| US2 | 1 | L221S |
| US3 | 1 | V109I |
| US5 | 1 | N96K |
| TRS | 1 | P327H |


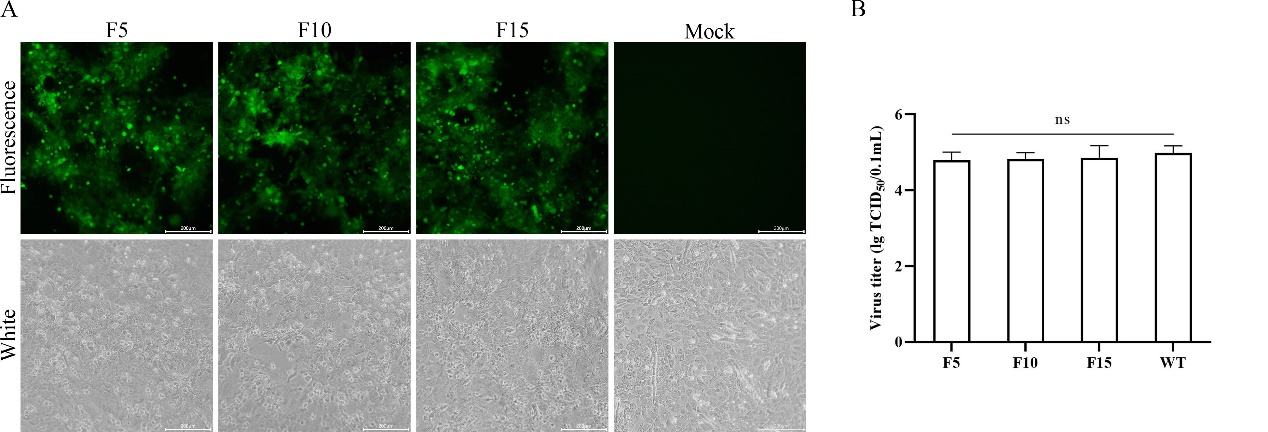


Figure S1: Genetic stability of recombinant virus XJ BAC. (A) The GFP expression levels and the cytopathic effect were observed in DEFs infected with the 5th, 10th, and 15th XJ BAC. (B) The viral titers of the 5th, 10th, and 15th XJ BAC were determined. No significant differences were observed among the three groups (ns, P > 0.05). The results were computed from three independent experiments.


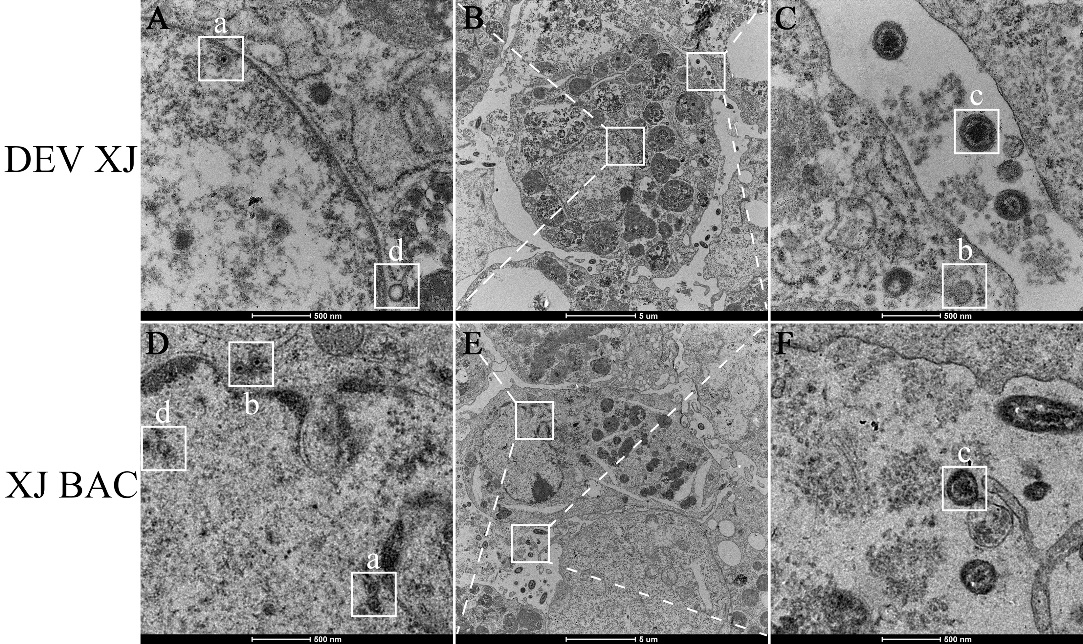


Figure S2: Ultrastructural morphologies of DEV XJ and XJ BAC. DEFs were infected with approximately 0.02 MOI of viruses and examined by electron microscopy. The white boxed areas in the middle images of DEV XJ (B) and XJ BAC (E) were enlarged on the left and right (A, C, D, and F). (a) Particles in the nucleus and the electron-dense core were wrapped in capsids. (b) Complete viral particles in the cytoplasm. (c) Mature viral particles were released out of cells. (d) Empty capsids caused by incorrect assembly were observed in the nucleus and cytoplasm.
